# A Multi-Sensor Data-Fusion Method Based on Cloud Model and Improved Evidence Theory

**DOI:** 10.3390/s22155902

**Published:** 2022-08-07

**Authors:** Xinjian Xiang, Kehan Li, Bingqiang Huang, Ying Cao

**Affiliations:** School of Automation and Electrical Engineering, Zhejiang University of Science and Technology, Hangzhou 310023, China

**Keywords:** sensor data fusion, cloud model, Dempster–Shafer evidence theory, cosine similarity, Hellinger distance

## Abstract

The essential factors of information-aware systems are heterogeneous multi-sensory devices. Because of the ambiguity and contradicting nature of multi-sensor data, a data-fusion method based on the cloud model and improved evidence theory is proposed. To complete the conversion from quantitative to qualitative data, the cloud model is employed to construct the basic probability assignment (BPA) function of the evidence corresponding to each data source. To address the issue that traditional evidence theory produces results that do not correspond to the facts when fusing conflicting evidence, the three measures of the Jousselme distance, cosine similarity, and the Jaccard coefficient are combined to measure the similarity of the evidence. The Hellinger distance of the interval is used to calculate the credibility of the evidence. The similarity and credibility are combined to improve the evidence, and the fusion is performed according to Dempster’s rule to finally obtain the results. The numerical example results show that the proposed improved evidence theory method has better convergence and focus, and the confidence in the correct proposition is up to 100%. Applying the proposed multi-sensor data-fusion method to early indoor fire detection, the method improves the accuracy by 0.9–6.4% and reduces the false alarm rate by 0.7–10.2% compared with traditional and other improved evidence theories, proving its validity and feasibility, which provides a certain reference value for multi-sensor information fusion.

## 1. Introduction

Heterogeneous multi-sensors play an important role in information perception, the acquired data may contain some ambiguous and conflicting information due to the limitations of multi-sensor devices’ measurement accuracy and the complexity of the working environment, which may result in inaccurate data-fusion decisions [1]. Consequently, the way to better handle multi-sensor data and improve data-fusion accuracy is a popular research direction in the field of data-fusion technology. Common data-fusion algorithms currently include Kalman filtering [2], Bayesian estimation [3], Dempster–Shafer (D-S) evidence theory [4], and artificial neural networks [5], etc. Bayesian networks and D-S evidence theory are commonly used to deal with the uncertainty in multi-sensor data, which frequently results in anomalous data. However, the Bayesian estimation fusion method requires access to prior data to generate new probability estimates, which is not always possible [6]. Dempster–Shafer (D-S) evidence theory is a theory of fuzzy reasoning proposed by Dempster in 1967 [7] and subsequently refined by Shafer [8]. It has been widely employed in areas such as target identification [9], multi-attribute decision analysis [10], fault diagnostics [11], and robotics research [12] due to its capacity to better handle uncertain and unknown situations with unknown prior probabilities. Although the D-S evidence theory has been applied in a number of fields, it has certain problems. One is that there is no unified method for determining the BPA function, and the other is that the evidence theory is prone to produce results that contradict the facts when dealing with highly conflicting evidence, and there is no unified method for solving this problem. Most scholars have done some research on the above two problems.

Determining the BPA function is an important step in evidence theory, which influences the accuracy of fusion results to some extent. Many researchers have proposed various methods for determining BPA functions [13,14,15]. The cloud model [16] is a concept proposed by Professor Li in 1995, which is a cognitive model based on probability statistics and fuzzy set theory. It can well portray the fuzziness and randomness of information and is applicable to the field of multi-sensor information fusion. Peng et al. [17] improved the multi-criteria group decision method by using a cloud-model method to deal with uncertain information based on information fusion and information measurement, Liu et al. [18] used the cloud model to describe the load direction in topology optimization with uncertainty, and Peng et al. [19] proposed an uncertain pure linguistic information multicriteria group decision-making method based on the cloud model, demonstrating the advantage of the cloud model in dealing with uncertain information. In this paper, the cloud model is used to determine the BPA function to convert measured quantitative data to qualitative concepts.

The directions for improving the accuracy of traditional evidence theory fusion can be divided into two major areas: improvement of combination rules [20,21] and improvement of the body of evidence. The former blames the D-S rule for producing results that contradict the facts, achieving certain results but destroying the D-S rule’s own advantages, such as the law of exchange and the law of union. The latter believes that the problem stems from the unreliability of the information source and uses an improved approach to the body of evidence to deal with the conflict, which retains the good characteristics of Dempster’s rule and weakens the influence of conflicting evidence on the fusion result. As Haenni [22] points out, the improvement of the body of evidence is more reasonable both from an engineering and mathematical standpoint. The calculation and assignment of weights to the body of evidence is critical to improving the body of evidence, and some scholars have conducted a series of studies on how to evaluate the body of evidence’s weights. Murphy [23] proposed a simple averaging method to assign the same weight to each piece of evidence, but it ignores the relationship between the evidence and is therefore unreasonable. Deng et al. [24] proposed a more convergent method based on the rules of evidence theory after weighted average processing of evidence based on trust degree, but it does not take into account the characteristics of the evidence itself. There are two methods for determining the weight of the body of evidence: according to the relationship between the evidence and according to the characteristics of the evidence itself. For the former, Wang et al. [25], Jousselme et al. [26], and Dong et al. [27] measure the relationship between evidence by using the Pignistic probability distance, the Jousselme distance, and cosine similarity, respectively; however, using a single measure of evidence relationship to find the weight of evidence does not accurately describe the relationship between evidence in certain cases. For the latter, scholars have proposed various uncertainty measures based on information entropy, such as Yager’s [28] dissonance measure based on the likelihood function and Deng’s [29] evidence uncertainty measure based on Shannon entropy, but such methods deal with evidence in a one-sided manner, replacing the entire uncertainty interval with only part of the evidence information. Deng et al. [30] developed a method for evaluating evidence uncertainty based on the Hellinger distance of the uncertainty interval, which is simple to compute and measures uncertainty well for a better integration effect. The relationship between evidence and the characteristics of the evidence itself do not affect each other and are both valid information available within the evidence, yet some current scholarly approaches to improving evidence theory consider only one of them to deal with the evidence, undermining the integrity of the evidentiary information. Some scholars have proposed ways to improve the evidence theory based on both, but they both have some room for improvement. For example, Tao et al. [31] proposed a multi-sensor data-fusion method based on the Pearson correlation coefficient and information entropy. Xiao et al. [32] proposed a multi-sensor data-fusion method based on belief dispersion of evidence and Deng entropy [29]. Wang et al. [33] combined the Jaccard coefficient and cosine similarity to calculate evidence similarity, combined with evidence-based precision and entropy of evidence to calculate evidence certainty. Although these methods combine the relationship between evidence and the characteristics of evidence itself, they all have certain disadvantages. The Pearson correlation coefficient is only used to portray the linear correlation between normally distributed attributes, which is more demanding on evidence. The Jaccard coefficient and cosine similarity sometimes cannot measure the relationship between evidence correctly. Using information entropy cannot measure the characteristics of evidence itself comprehensively, etc.

In order to more accurately measure the relationship between evidence and the characteristics of evidence itself, and improve the accuracy of data fusion, this paper proposes an improved evidence-theory method based on multiple relationship measures and focal element interval distance. We combine the Jousselme distance, cosine similarity and the Jaccard coefficient to calculate the similarity between the evidence, and we use the Hellinger distance between the evidence determination intervals to measure the certainty of the evidence. Based on these calculations, the evidence weight coefficients are then jointly improved. Finally, the original evidence is average-weighted and fused by using the Dempster rule to produce the result. In addition, we analyze the results of the arithmetic examples to demonstrate the validity of the proposed improved evidence theory. By using the aforementioned improved evidence theory along with cloud model, we developed a multi-sensor data-fusion method. The BPA functions corresponding to each data source are determined based on the cloud model, which converts the collected quantitative data into stereotypical concepts. The fusion results are obtained by fusing each BPA function by using the improved evidence theory mentioned above.

Multi-sensor data-fusion technology can combine relevant information within multiple sensors, thereby increasing the safety and reliability of the overall system. The proposed multi-sensor data-fusion method can be utilized in multi-sensor systems in various fields, such as fault-determination systems, target identification systems, environmental monitoring systems, and intelligent firefighting systems, among others. Due to external factors or their own aging faults, one or more sensors may acquire incorrect information, causing the fusion results to be contradictory to the facts. The proposed method overcomes the problem, improves the handling of ambiguity in sensor data, increases the reliability of data fusion results, and makes it easier for people to make appropriate decisions. We establish an early indoor fire detection model to test the efficacy of the proposed strategy. The proposed method improves accuracy by 0.7–10.2% and reduces false alarm rate by 0.9–6.4% when compared to the traditional evidence theory and other improved evidence theories. It has better fusion performance, which provides some reference value for multi-sensor data fusion.

## 2. Preliminaries

This section provides a brief overview of D-S evidence theory and the cloud model.

### 2.1. Cloud Model

Let *X* be a quantitative domain (X={x}) and *U* be a qualitative concept on the domain *X*. For any element x(x∈X) and *x* is a single random realization on *U*, the certainty of x to *U* is y(x)∈[0,1], which is a random number with stable tendency, the distribution of *x* over the domain *X* is called a cloud model and each (x y(x)) becomes a cloud drop [34].

The cloud model completes the conversion of quantitative data to qualitative concepts through numerical characteristic expectation ($E_{x}$), entropy ($E_{n}$), and hyperentropy ($H_{e}$), where expectation is the expected value of the distribution of cloud droplets in the theoretical domain, entropy reflects the dispersion of cloud droplets, and hyperentropy reflects the dispersion of entropy. Because the values of the characteristics corresponding to the evaluation indices have some stability across the multi-sensor domain and the interval distributions generally follow a normal distribution that is more realistic, the normal cloud model is used in this research. Each parameter’s computation formula is presented in Equation (1),

(1) $$\{\begin{array}{c} E_{xij}=\frac{C_{ij,max}+C_{ij,min}}{2}\\ E_{nij}=\frac{C_{ij,max}-C_{ij,min}}{2.355}\\ He=\lambda_{i} \end{array},$$

where $\left[C_{ij,min},C_{ij,max}\right]$ are the range of values of the evaluation interval corresponding to the *j*th certain evaluation index inside the *i*th data type of the multi-sensor system, and $\lambda_{i}$ is a value determined by experts based on experience and is typically 0.01. It is worth noting that the maximum and lower bounds of each data source’s evaluation value are the expectation of both cloud $E_{x}$ values. The entropy of the traditional cloud model is $E_{nij}=(C_{ij,max}-C_{ij,min})/6$, when the data is near the endpoint value, and the corresponding degree of certainty tends to 0. However, the endpoint value of the interval divided by each level is the transition boundary value of the two adjacent levels, and the edge value should belong to the upper and lower intervals at the same time. Therefore, in order to represent the boundary ambiguity of adjacent ranks, the divisor for finding the entropy is determined to be 2.355.

Let ($E_{xij},E_{nij},H_{e})$ be the three numerical properties of a cloud for a given one-dimensional domain, and the procedure for this one-dimensional normal cloud generator is:

1. Generate a normal random number $E_{nij}^{\prime }$ with $E_{nij}$ as the expected value and $H_{e}{}^{2}$ as the variance.
2. Generate a normal random number $x_{ij}$ with $E_{xij}$ as the expected value and $E_{nij}^{\prime }{}^{2}$ as the variance.
3. Calculate $y_{ij}=\exp\left(-\frac{{\left(x_{ij}-E_{xij}\right)}^{2}}{2E_{nij}^{\prime }{}^{2}}\right)$, where $x_{ij}$ is a specific quantified value, $y_{ij}$ is the degree of determination of $x_{ij}$ on qualitative index *U*, and ($x_{ij},\;y_{ij}$) is the cloud drop.
4. Repeat the above steps until *N* cloud drops are generated.

### 2.2. Dempster–Shafer Evidence Theory

Let $\Theta =\theta_{1},\theta_{2},\ldots ,\theta_{n}$ be a finite identification framework in the D-S evidence theory, where $\Theta =\theta_{1},\theta_{2},\ldots ,\theta_{n}$ are all possible events and $\theta_{i}\left(i=\left[1,n\right]\right)$ is a subset of the recognition frame Θ. The underlying trust function *m* be a mapping from the set $2^{\Theta }\to \left[0,1\right]$, with *A* being any subset of Θ and it satisfies

(2) $$\{\begin{array}{c} m\left(\emptyset \right)=0\\ \sum_{A\subset \Theta }m\left(A\right)=1 \end{array}$$

We call *m* the basic probability assignment function (BPA function for short) of Θ  [35], where m(∅) denotes the degree of confidence of the evidence in the empty set. If *m*(*A*) > 0, then *A* is called a focal element within the identification framework Θ, and *m*(*A*) reflects the degree of trust of the evidence in *A*. In particular, the condition m(∅)=0 is not necessarily satisfied. For the open evaluation set space, m(∅) is not necessarily equal to 0. In this paper, we only consider the case in the closed evaluation set space.

For recognition framework $\Theta =\theta_{1},\theta_{2},\ldots ,\theta_{n}$ and BPA function *m*(*A*), *Bel*(*A*) is defined as the confidence function, which is the sum of the potential probability assignments of all subsets of *A*, indicating the degree of certainty of the proposition *A*, as shown in Equation (3):

(3) $$Bel\left(A\right)=\sum_{B\subset A}m\left(B\right),\forall A\subset \Theta \;.$$

*Pl*(*A*) is the likelihood function of *A*, as defined in Equation (4), indicates the degree of trust that does not deny *A*,

(4) $$Pl\left(A\right)=1-Bel\left(\bar{A}\right)=\sum_{B\cap^{\;}A\neq \emptyset }m\left(B\right).$$

**Figure 1:**
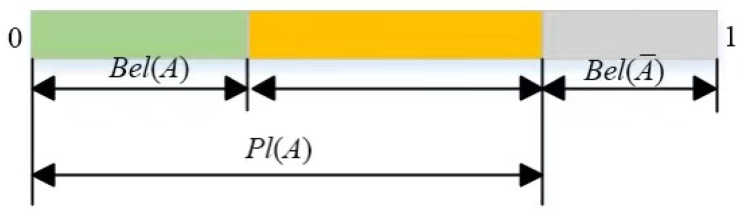
Diagram of evidence intervals.

The intervals of evidence are shown in Figure 1, where [0,Bel(A)] is the support interval of proposition *A*, [Bel(A),Pl(A)] is the uncertainty interval of proposition *A*, and [Pl(A),1] is the rejection interval of evidence. Among them, support interval and rejection interval together constitute the definite interval of evidence, which can represent the certainty of evidence.

Let $m_{1}$ and $m_{2}$ be two BPA functions on the same finite identification frame Θ, with focal elements $B_{1},B_{2},\ldots ,B_{n}$ and $C_{1},C_{2},\ldots ,C_{n}$. Then the D-S evidence theory combination rule rules are as follows in Equations (5) and (6):

(5) $$m\left(A\right)=\{\begin{array}{c} \begin{array}{c} \frac{1}{1-K}\sum_{B_{i}\cap^{\;}C_{j}=A}m_{1}\left(B_{i}\right)m_{2}\left(C_{j}\right)\;,\;A\neq \emptyset \end{array}\\ 0\;\;\;\;\;\;\;\;\;\;\;\;\;\;\;\;\;\;\;\;\;\;\;\;\;\;\;\;\;\;\;\;\;\;\;\;\;\;\;\;\;\;\;\;\;\;\;\;\;\;\;\;\;\;,\;A=\emptyset \end{array}$$

(6) $$K=\sum_{B_{i}\cap^{\;}C_{j}=\emptyset }m_{1}\left(B_{i}\right)m_{2}\left(C_{j}\right),$$

where *K* is the coefficient of evidence conflict between $m_{1}$ and $m_{2}$, the higher *K* value indicates the greater the degree of evidence conflict, and the values of *K* range from 0 to 1. 

## 3. The Proposed Method

Based on the above theoretical knowledge, this paper proposes a heterogeneous data-fusion method based on a cloud model and improved evidence theory. In order to obtain the BPA function of evidence more accurately, we consider the ambiguity of multi-sensor data when completing data transformation by using the cloud model. To improve the reliability of the fusion results, we propose a new method for measuring the similarity of evidence and improve the evidence by combining the similarity and certainty of evidence together. The specific method for determining the BPA function and calculating the similarity of evidence and the certainty of evidence are described in this section, and finally the overall steps of the method are proposed.

### 3.1. Determination Method of BPA Function

It is assumed that the multi-sensor system’s data information is pre-processed to extract *n* classes of data, forming *n* bodies of evidence, i.e., $X=\left(x_{1},x_{2},x_{3},\ldots ,x_{n}\right)$, where $x_{i}\left(i=\left[1,n\right]\right)$ is the *i*th class of data measured by the system. Based on the knowledge gained from the cloud model, the membership degree $\mu_{ij}(k)$ for the values of discrete feature variables is calculated as follows in Equation (7):

(7) $$\mu_{ij}\left(k\right)=e^{-\frac{{\left(x_{i}-E_{xij}\right)}^{2}}{2E_{nij}^{\prime }{}^{2}}},$$

where $\mu_{ij}\left(k\right)$ is the membership of the *i*th class of data relative to the *j*th evaluation index under the *k*th judgment within the same acquisition cycle of the multi-sensor system, $E_{xij}$ is the expectation value of class *i* data relative to the *j*th evaluation index obtained in Equation (1), and $E_{nij}^{\prime }$ is a normal random number generated with $E_{nij}\;$ as the expectation and $H_{e}$ as the standard deviation obtained in Equation (1).

*k* is the number of times the multi-sensor acquires data in the same acquisition cycle, when *k* is greater than 1, the membership of class *i* data with respect to the *j*th evaluation index can be determined by the maximum of the *k* affiliation values when the feature parameters have multiple values:

(8) $$\mu_{ij}=\max\left(\mu_{ij}\left(a\right)\right),a=1,2,\ldots ,k.$$

The multi-sensor membership matrix can be calculated based on the membership degree $\mu_{ij}$:

(9) $$R_{n\times m}=\left(\begin{array}{cc} \begin{array}{cc} \mu_{11} & \mu_{12}\\ \mu_{21} & \mu_{22} \end{array} & \begin{array}{cc} \ldots & \mu_{1m}\\ \ldots & \mu_{2m} \end{array}\\ \begin{array}{cc} \vdots & \vdots \\ \mu_{n1} & \mu_{n2} \end{array} & \begin{array}{cc} \vdots & \vdots \\ \ldots & \mu_{nm} \end{array} \end{array}\right).$$

The elements in each row in Equation (9) represent the membership of the *i*th (*i* = 1, 2, ⋯, *n*) class of data of the multi-sensor for the *j*th (*j* = 1, 2, ⋯, *m*) evaluation index, and the elements in each column represent the membership of all data information collected by the multi-sensor system at a certain time for the *j*th (*j* = 1, 2, ⋯, *m*) evaluation index.

The obtained membership matrix $R_{n\times m}$ basically satisfies the definition of probability assignment but does not satisfy $\sum_{j=1}^{m}\mu_{ij}=1$. Considering that the actual use of the sensor will produce a certain measurement error, the following definition is added to transform the membership of each evaluation index into a BPA function:

(10) $$\{\begin{array}{c} \gamma_{i}=1-\max\left(\mu_{i1,}\mu_{i2},\ldots ,\mu_{im}\right)\\ m_{i}\left(\Theta \right)=\gamma_{i}\\ m_{i}\left(A_{j}\right)=\left(1-\gamma_{i}\right)\frac{\mu_{ij,}}{\sum_{j=1}^{m}\mu_{ij}} \end{array},$$

where $\gamma_{i}$ denotes the uncertainty of the *i*th characteristic parameter, $m_{i}\left(\Theta \right)$ is the basic probability assignment value of the uncertainty of the *i*th piece of evidence, and $m_{i}\left(A_{j}\right)$ is the basic probability assignment value of the *j*th evaluation index of the *i*th piece of evidence.

### 3.2. Similarity of Evidence

Classical measures for describing the relationship between evidence include: conflict coefficient *K*, Pignistic probability distance, Jousselme distance and cosine similarity, and so on. The computation of the conflict coefficient *K* is given in (6), and assuming that the evidence bodies $m_{1}$ and $m_{2}$ are BPA functions of the identification framework $\Theta =\theta_{1},\theta_{2},\ldots ,\theta_{n}$, the calculation of the Pignistic probability distance, Jousselme distance, and cosine similarity is given below.

1. Pignistic probability distance [25]

Pignistic probability distance is a measure of conflicting relationships between evidence. Let the recognition frame $\Theta =\theta_{1},\theta_{2},\ldots ,\theta_{n}$, *m* is the BPA function of Θ, and if A⊆Θ, then

(11) $$BetP_{m}\left(A\right)=\sum_{B\subseteq \Theta }\frac{\left|A\cap B\right|}{\left|B\right|}m\left(B\right)\;$$

is said to be the Pignistic probability of the focal element *A*.

Assuming that $BetP_{m_{1}}$ and $BetP_{m_{2}}$ are the corresponding Pignistic probability functions, the Pignistic probability distances are calculated as follows:

(12) $$difBetP_{m_{2}}^{m_{1}}=max_{A\subseteq \Theta }{\left(\left|BetP_{m_{1}}\left(A\right)-BetP_{m_{2}}\left(A\right)\right|\right)}_{.}$$

2. Jousselme distance [26]

(13) $$d_{BPA}\left(m_{1},m_{2}\right)=\sqrt{\frac{1}{2}{\left(m_{1}-m_{2}\right)}^{T}D\left(m_{1}-m_{2}\right)},$$

where $m_{1}$ and $m_{2}$ are the vector forms of the evidences $m_{1}$ and $m_{2}$, and ***D*** is a $2^{\Theta }\times 2^{\Theta }$ positive definite matrix, its mathematical expression is: $D=\left(\begin{array}{cc} \begin{array}{cc} d_{11} & d_{12}\\ d_{21} & d_{22} \end{array} & \begin{array}{cc} \ldots & d_{1n}\\ \ldots & d_{2n} \end{array}\\ \begin{array}{cc} \vdots & \vdots \\ d_{n1} & d_{n2} \end{array} & \begin{array}{cc} \vdots & \vdots \\ \ldots & d_{nn} \end{array} \end{array}\right)$, where the element $d_{ij}=J\left(\theta_{i},\theta_{j}\right)=\frac{\left|\theta_{i}\cap^{\;}\theta_{i}\right|}{\left|\theta_{i}\cup^{\;}\theta_{j}\right|}$, $\theta_{i}$ is any focal element in evidence $m_{1}$ and $\theta_{j}$ is any focal element in evidence $m_{2}$, which can also be called the Jaccard coefficient and can be used to reveal the relationship between unifocal and multifocal elements of the evidence.

The Jousselme distance is a measure of the conflicting relationships of the evidence, and the higher its value, the greater the conflict between the evidence.

3. Cosine similarity [27]

The cosine similarity can be used to calculate the similarity of the evidence. The greater the cosine similarity, the greater the confidence between the evidence.

(14) $$\cos\left(m_{1},m_{2}\right)=\frac{m_{1}\cdot m_{2}^{T}}{\left|\left|m_{1}\right|\right|\cdot \left|\left|m_{2}\right|\right|},$$

where $\left|\left|m_{i}\right|\right|=\sqrt{m_{i}\cdot m_{i}^{T}}$.

The accuracy of the various measurements is examined based on the above computation by calculating the measures under different conditions in conjunction with Example 1.

**Example** **1.** Suppose there are identification frames Θ={a,b,c,d} with different distributions of evidence bodies under different conditions, as shown in Table 1.

The body of evidence under Situation 1 is identical, and its conflict coefficient K is calculated by using Equation (6), yielding 0.75, which contradicts the fact, whereas cosine similarity and the Jousselme distance yield 1, which is consistent with the fact. Situation 2’s evidence is radically different, and the Jousselme distance metric produces 0.707, which is inconsistent with the facts, whereas the cosine similarity computation yields 0, which is consistent with the facts. Because it is impossible to determine whether the body of evidence $m_{2}$ under Situation 3 supports each focal element on average, the body of evidence under Situation 3 is somewhat conflicting, and the results of the Pignistic probability distance and cosine similarity are both 0, which contradict the facts, the result of the Jousselme distance is 0.577, which is more consistent with the facts. 

From the above analysis, the cosine similarity measure is more accurate when measuring evidence with only a subset of single focal elements, and less accurate when faced with evidence containing a subset of multiple focal elements. Wang et al. [33] combined cosine similarity and the Jaccard coefficient to measure the relationship between evidence. But both measures are similarity measures, and the analysis of how the evidence relates to each other is not thorough enough. This can lead to inaccurate measurements in some situations, such as when evidence $m_{1}$ and $m_{2}$ in Situation 4 point to different correct propositions and there is a big disagreement. However, Wang’s method gives a similarity of 0.80, which is less consistent with the facts. Therefore, this paper proposes to combine conflicting evidence and similarity to jointly measure the relationship between evidence. Because the Jousselme distance can measure the relationship between evidence more accurately in most cases, and it is introduced to jointly measure the relationship between evidence.

Assuming the identification framework $\Theta =A_{1},A_{2},\ldots ,A_{n}$, we define the local similarity of evidence $s_{ij}$ as:

(15) $$\{\begin{array}{c} J\left(A_{a},A_{b}\right)=\frac{\left|A_{a}\cap A_{b}\right|}{\left|A_{a}\cup A_{b}\right|},\forall A_{a},A_{b}\subseteq \Theta \\ s_{ij}=\left(1-d_{BPA}\left(m_{1},m_{2}\right)\right)\times \frac{\sum_{a=1}^{n}\sum_{b=1}^{n}m_{i}\left(A_{a}\right)m_{j}\left(A_{b}\right)\times J\left(A_{a},A_{b}\right)}{\sqrt{\sum_{c=1}^{n}m_{i}{\left(A_{c}\right)}^{2}}\sqrt{\sum_{c=1}^{n}m_{j}{\left(A_{c}\right)}^{2}}} \end{array}$$

According to Equation (15), the local similarities of the evidence under different situations in Example 1 are: 1, 0, 0.244, and 0.470, all of which are more consistent with the facts. Based on the local similarity $s_{ij}$, the global similarity $s_{i}$ can be derived for each piece of evidence, and its normalization can lead to the similarity-based weight coefficient $\alpha_{i}$, which is calculated as follows:

(16) $$\{\begin{array}{c} s_{i}=\sum_{j=1,i\neq j}^{n}s_{ij}\\ \alpha_{i}=\frac{s_{i}}{\sum_{j=1}^{n}s_{j}} \end{array}.$$

### 3.3. Certainty of Evidence

The properties of the evidence itself can be measured based on the degree of certainty of the evidence. In probability theory, the Hellinger distance is a complete distance metric defined in the space of probability distributions and can be used to measure the similarity between two probability distributions. It has the advantage of stability and reliability compared to other metrics. Deng et al. [30] measured the uncertainty of the evidence itself by calculating the uncertainty interval distance of the evidence focal elements. However, finding the weight of the evidence based on uncertainty involves more steps and is more tedious than finding the weight based on certainty, so this paper proposes a method by which to combine the Hellinger distance of the evidence support interval and rejection interval to jointly measure the certainty of the evidence.

Suppose $X=\left\{x_{1},x_{2},\ldots x_{n}\right\}\;and\;Y=\left\{y_{1},y_{2},\ldots y_{n}\right\}$ are two probability distribution vectors of the random variable Z, and the Hellinger distance is

(17) $$Hel(X||Y)=\sqrt{\frac{1}{2}\sum_{i=1}^{n}{\left(\sqrt{x_{i}}-\sqrt{y_{i}}\right)}^{2}}.$$

Assuming the identification framework $\Theta =A_{1},A_{2},\ldots ,A_{n}$ and defining $DU\left(m_{i}\right)$ as the evidence certainty, the calculation of $DU\left(m_{i}\right)$ is as follows:

(18) $$DU\left(m_{i}\right)=\sum_{j=1}^{n}\sqrt{2}\times \left(\sqrt{\frac{1}{2}\times \left[{\left(\sqrt{Bel\left(m_{i}\left(A_{j}\right)\right)}-0\right)}^{2}+{\left(1-\sqrt{Pl\left(m_{i}\left(A_{j}\right)\right)}\;\right)}^{2}\right]}\right)\;,$$

where $\sqrt{2}$ is the normalization factor. The Hellinger distance reaches its maximum when the evidence determines that the interval is [1,1] or [0,0], which leads to the calculation of the normalization factor: $\frac{1}{Hel\left[\left[1,1\right],\left[0,1\right]\right]}=\sqrt{2}$.

Normalizing the resulting determinacy $DU\left(m_{i}\right)$ obtains the weight of the evidence based on the determinacy:

(19) $$\beta_{i}=\frac{DU\left(m_{i}\right)}{\sum_{j=1}^{n}DUm_{j}}.$$

### 3.4. Steps of the Proposed Method

**Figure 2:**
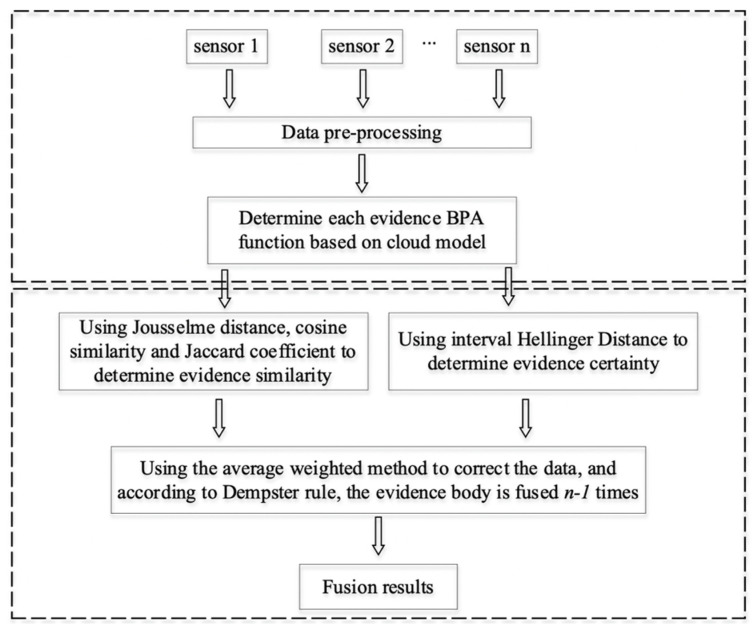
Flow chart of the proposed method.

Based on the above study, the specific steps of the proposed method in this paper are given as follows, and the flow chart is shown in Figure 2.

**Step 1:** After pre-processing the data from sensors, the BPA function of each data source related to the body of evidence is calculated by integrating the cloud model and each data evaluation index.
**Step 2:** With the obtained BPA function of each evidence, the weight $\alpha_{i}$ based on the similarity of evidence is calculated by combining Equations (15) and (16), and the weight $\beta_{i}$ based on the certainty of evidence is calculated by combining Equations (18) and (19).
**Step 3:** With the weights $\alpha_{i}$ and $\beta_{i}$, the total weight of the evidence body is calculated and normalized to obtain the final weight $\omega_{i}$, which is calculated as follows:
  (20) $$\{\begin{array}{c} \omega_{i}^{\prime }=\alpha_{i}\times \beta_{i}\\ \omega_{i}=\frac{\omega_{i}^{\prime }}{\sum_{j=1}^{n}\omega_{j}^{\prime }} \end{array}.$$
**Step 4:** Based on the weights $\omega_{i}$, the original evidence is averaged and weighted to obtain the processed body of evidence *m*,
  (21) $$m\left(A\right)=\sum_{i=1}^{n}\omega_{i}\times m_{i}\left(A\right).$$
**Step 5:** Use Dempster’s fusion rule to perform *n* − 1 fusion for evidence body *m*.

## 4. Numerical Example and Simulation Results

In this section, the proposed improved D-S evidence theory method based on similarity and certainty, as well as the proposed overall method of heterogeneous data fusion based on cloud model and evidence theory, are evaluated and simulated to demonstrate the feasibility and effectiveness of the proposed method in this paper.

### 4.1. The method for Improving D-S Evidence Theory

In this section, four common conflicting, normal, and multi-quantity single-focal and multi-focal element evidences are fused based on the proposed improvement method, comparing traditional evidence theory, classical improvement methods, and similar improvement methods, and demonstrating the effectiveness of the proposed methods in this paper through Examples 2–4. We take the methods proposed by Deng Z. [30] and Wang [33] as similar improvement methods.

**Example** **2.** In evidence theory, there are four common sorts of conflicts: complete conflict, 0 trust conflict, 1 trust conflict, and severe conflict [36], and the BPA functions for the four typical conflicts are provided in Table 2.

**Figure 3:**
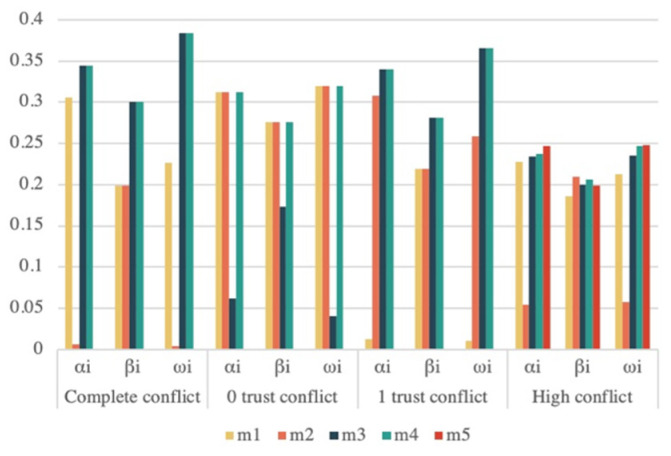
Four common types of conflicting evidence weights.

The global similarity $s_{i}$ and own determination $DU\left(m_{i}\right)$ of each evidence under the four conflict types are shown in Table 3. The weights $\alpha_{i}$ and $\beta_{i}$ of the evidence can be calculated based on the degree of similarity $s_{i}$ and the degree of certainty $DU\left(m_{i}\right)$, and the overall weight $\omega_{i}$ of the evidence can be obtained by combining the weights $\alpha_{i}$ and $\beta_{i}$. Figure 3 displays the distribution chart for each weight. Figure 3 shows that the weights of conflicting evidence are lower than those of normal evidence, and the distribution of each weight is consistent with the facts. We combined similarity and certainty to improve the body of evidence in order to improve the science of data fusion, and it should be noted that because the certainty of evidence describes the characteristics of the evidence itself, which includes the interval information of all focal elements within the evidence and is independent of the relationship between the evidence, the weights $\alpha_{i}$ and $\beta_{i}$ are not always positively correlated.

**Figure 4:**
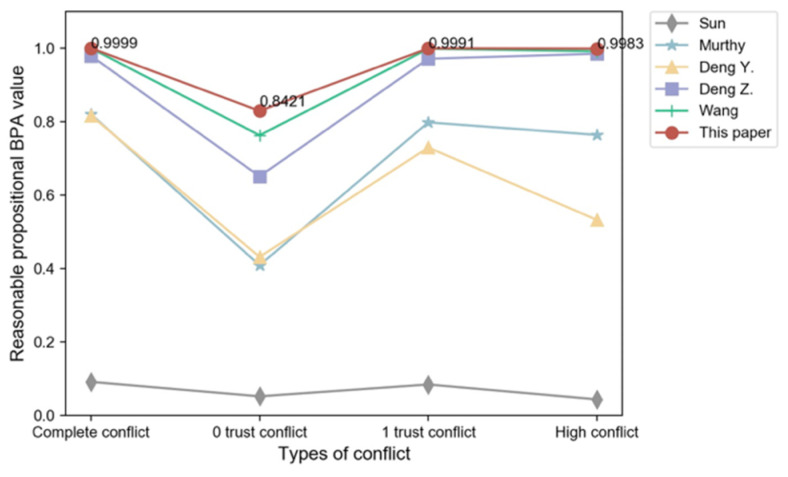
Comparison of reasonable proposition BPA value of different fusion algorithms.

Table 4 displays the fusion results of the traditional D-S rule, the methods proposed by Sun [20], Murphy [23], Deng Y. [24], Deng Z. [30], and Wang [33], and the improved method proposed in this paper. As seen in Table 4, when confronted with the four conflicting situations listed above, the D-S fusion rule fails or does not match the genuine situation, and Sun’s method allocates the uncertainty to the entire set, resulting in high BPA values for the entire set that do not fit the true situation. The larger the value of BPA after fusing, the greater the amount of confidence in the proposition. Although the approaches of Murphy, Deng Y., Deng Z., and Wang yield correct answers, the method proposed in this work yields a higher BPA function value and converges faster, demonstrating that the improved method in this research performs better than the other methods in resolving the four conflicts. The fusion BPA results on the reasonable propositions of each algorithm are shown in Figure 4.

**Example** **3.** Assume the radar identification library contains three radar model data *A*, *B*, and *C*, with identification frame Θ={A,B,C,AC}. Five existing heterogeneous sensors are used separately to identify the radar radiation sources, yielding a total of five conflicting evidences ranging from *m*_1_ to *m*_5_. Table 5 and Table 6 show the results of a specific two times, which represent the data distribution of multi-quantity single and multi-focal element conflict evidence, respectively.

**Figure 5:**
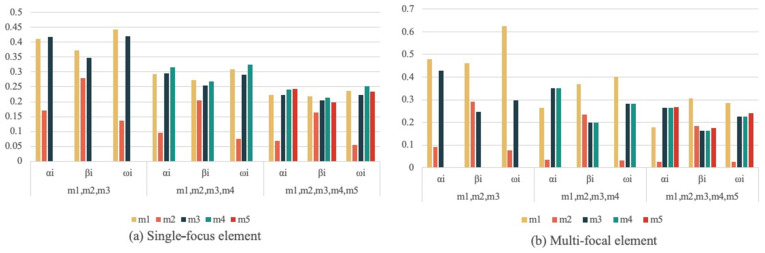
Weight of evidence under different amounts of evidence. (**a**) Single-focal element evidence weights. (**b**) Multi-focal element evidence weights.

The global similarity $s_{i}$ and certainty $DU\left(m_{i}\right)$ of each evidence under single and multifocal elements are shown in Table 7. The weights of evidence $\alpha_{i}$, $\beta_{i},$ and $\omega_{i}$ for a different number of evidence cases are shown in Figure 5. From Figure 5, it can be seen that the weight of conflicting evidence is less than the normal weight, the weight occupied by conflicting evidence gradually decreases as the number of evidence increases, and the distribution of each evidence is consistent with the facts, which proves the rationality of the method proposed in this paper.

**Figure 6:**
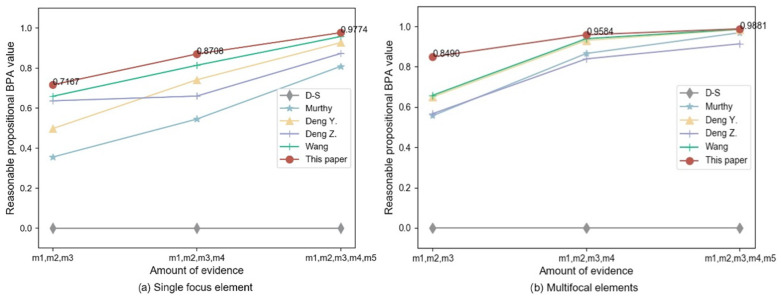
Comparison of the fusion results of multiple evidence. (**a**) Single focus element evidence fusion results. (**b**) Multi-focal element evidence fusion results.

To verify the effectiveness of the improved method proposed in this paper, the evidences are fused by using Murthy [23], Deng Y. [24], Deng Z. [30], and Wang [33], and the proposed method are fused respectively. Table 8 shows the fusion results for each method, and Figure 6 shows the comparison of BPA values for reasonable propositions. From the fusion results and comparison results, it can be concluded that when facing different numbers of single and multifocal element conflicting evidence bodies, the traditional D-S fusion results all contradict the facts. Although Murthy [23], Deng Y. [24], Deng Z. [30], and Wang [33] and the proposed method all point to the correct results, the BPA functions of the proposed method are higher than the other two improved methods, and as the number of evidence bodies increases, the improved method converges faster with higher accuracy on the BPA value as the number of evidence bodies increases.

**Example** **4.** With the identification frame Θ={A,B,C}, there are five normal evidence bodies from m_1_ to m_5_, and the distribution is shown in Table 9.

The proposed improved method’s fusion of normal evidence is compared to the traditional evidence theory to demonstrate the proposed improved method’s superior performance in dealing with normal data, and the fusion results are shown in Table 10. Compared to the traditional evidence theory algorithm, the proposed method can also get correct results when dealing with normal body of evidence and has a higher BPA function with higher credibility.

According to the above examples, the similarity and certainty-based evidence theory fusion algorithm proposed in this paper performs better in handling both normal and conflicting evidence bodies, demonstrating the improved method’s rationality and effectiveness.

### 4.2. The Proposed Holistic Approach

To demonstrate the feasibility and effectiveness of the proposed data-fusion method, the heterogeneous data-fusion method combining cloud model and the proposed improved evidence theory in this paper is used for indoor early fire detection in this subsection.

**Figure 7:**
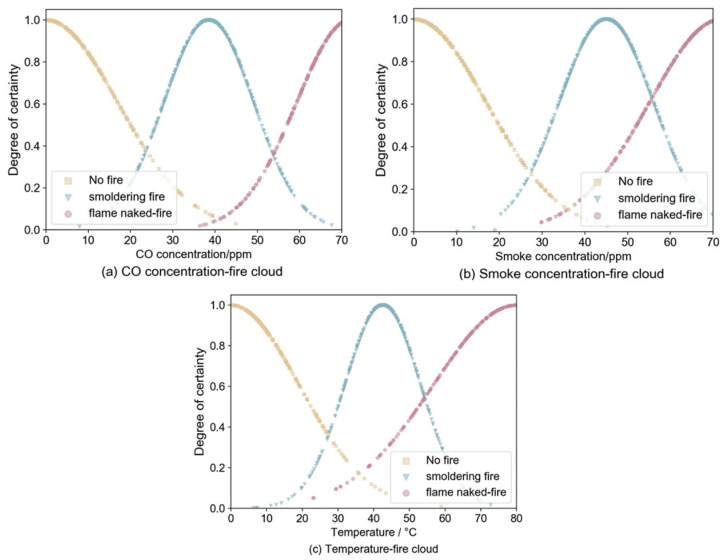
Fire characteristic parameter cloud chart. (**a**) CO concentration-fire cloud chart. (**b**) Smoke concentration-fire cloud chart. (**c**) Temperature-fire cloud chart.

It has been discovered that the combination of temperature, smoke concentration, and CO concentration has superior detection performance in fires [37], and the above information is collected as fire characteristic parameters in this paper. The fire discrimination results are divided into three categories: no fire, smoldering fire, and open fire. In the established fire identification framework $\Theta =\left\{\theta_{1},\theta_{2},\theta_{3},\theta_{1}\theta_{2}\theta_{3}\right\}$, $\theta_{1},\theta_{2},\theta_{3}$ represent no fire, smoldering fire, and open fire, respectively, and $\theta_{1}\theta_{2}\theta_{3}$ indicates uncertainty of fire. Lin et al. [38] proposed a fire-detection method by using the Jousselme distance to improve the evidence corresponding to the fire characteristic parameters and fusing the evidence according to Dempster’s rule to improve the timeliness of detection. However, the method ignored the characteristics of the evidence body itself and did not fully exploit the fire data information. Because the attribute values corresponding to the three fire characteristic parameters of CO concentration, smoke concentration, and temperature have certain stability and the interval distribution obeys normal distribution within a certain value interval [39], the cloud model of each data source based on the forward cloud generator and the evaluation index of each parameter is built, and the cloud diagram is shown in Figure 7.

PyroSim fire simulation software provides a visual user interface for fire dynamic simulation (FDS) and can more accurately predict the distribution of characteristic parameters such as fire smoke and temperature [40], so this paper simulates the occurrence of fire to obtain fire characteristic parameters. We build the indoor scenario as follows:

- The length, width and height of the room are 5, 5, 3 m;
- The room has a sofa, wooden bed and wooden table, in the upper left corner of the room from the wall 1 m set CO, temperature and smoke sensor group;
- Set the vent: room left wall with 1 × 1 m window, room directly opposite the sofa with 1.2 × 2 m door;
- The fire burning material is n-Heptane, the center of combustion is the center of the wooden bed, the burning area is 1 m^2^.

By setting different heat release rate and heat ramp up time to simulate the occurrence of open fire and negative fire in the room, the starting room temperature is 30 °C, the simulation time is 30 s, and the data acquisition frequency is 2 Hz. Based on the proposed data-fusion method, a fire detection model is built. The initial fire detection probability is estimated by combining CO concentration, smoke concentration, and temperature data. The probability of smoldering fire and open flame within the initial fire detection probability is also added, and if it is greater than 0.75, the fire occurred in the room. 

**Figure 8:**
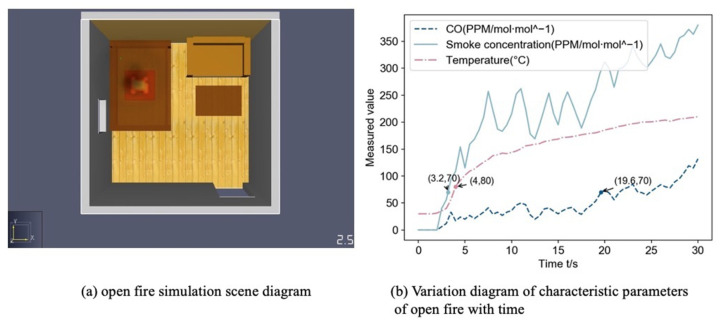
Open fire simulation information diagram.

Figure 8a depicts a simulation of an open fire with visible fire and black smoke visible at t = 2.5 s. Figure 8b shows the change of the measured CO concentration, smoke concentration, and temperature data with time. When the probability of an open fire is 1, the values of CO, smoke, and temperature are the thresholds, and the time when each parameter first reached the threshold is shown in Figure 8b. The three characteristic parameters of CO, smoke, and temperature had almost no fluctuation in the first 2 s and increased rapidly after 2 s. The temperature and smoke reached the threshold value relatively quickly, and all parameters showed an increasing trend in the first 30 s response time.

**Figure 9:**
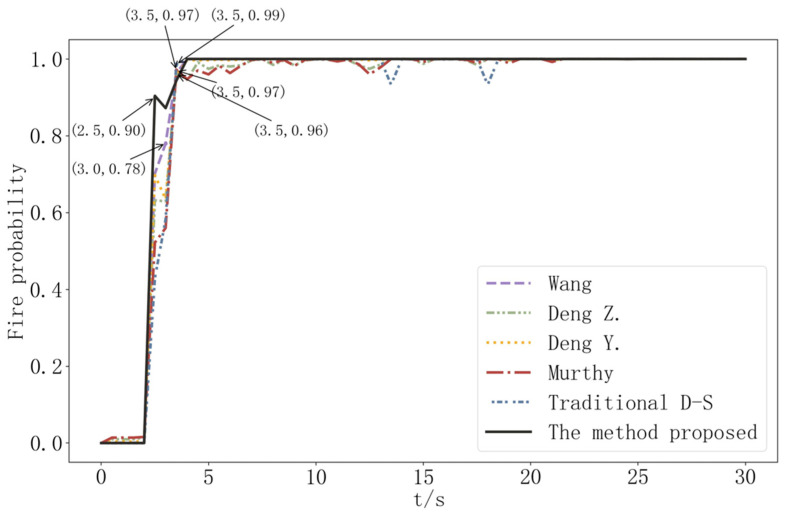
Fire occurrence probability comparison in open fire scene.

To determine whether a fire has occurred, the early open fire data from this simulation are fused using the traditional D-S evidence theory, the methods proposed by Murphy [23], Deng Y. [24], Deng. Z. [30], and Wang [33] and this paper, respectively. Because the frequency of data acquisition in the simulation is 2 Hz, the period of data fusion is 0.5 s. The traditional evidence theory, Murphy’s Deng Y’s., and Deng. Z’s methods all detect fire at t = 3.5 s, Wang’s method detects fire at t = 3 s, and the proposed method detects fire at t = 2.5 s, proving the method’s effectiveness. Figure 9 depicts the probability comparison of fire occurrence in this open fire scenario.

**Figure 10:**
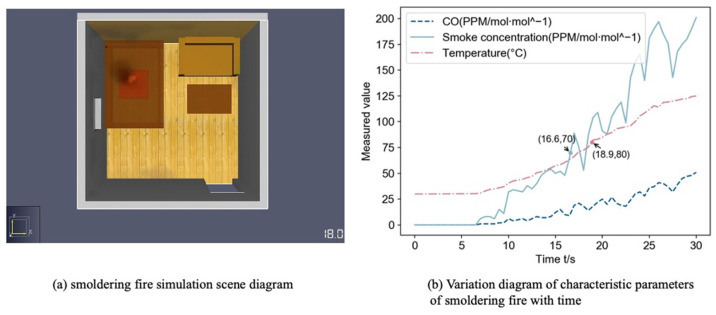
Smoldering fire simulation information diagram.

A smoldering fire’s combustion features include the emission of a significant amount of black smoke from the combustion point prior to the appearance of the evident fire. Figure 10a depicts a simulation of a smoldering fire, with a clear fire visible at t = 18 s. Figure 10b displays a time-plot of the data collected by the multi-sensor group during the first 30 s. As shown in Figure 10b, the rising trend of each characteristic parameter in the shaded fire scenario is slower than it is in the open fire scenario, and the parameters only continue to grow after 7 s as a result of the early shaded fire’s insufficient combustion.

**Figure 11:**
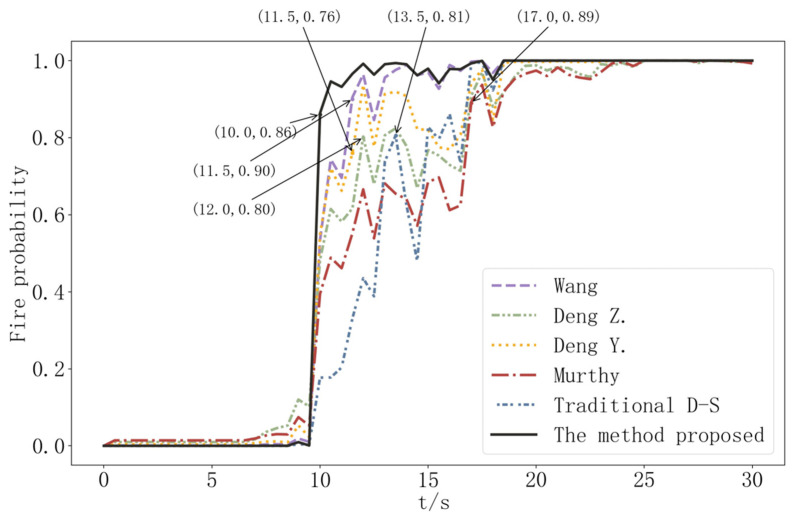
Fire occurrence probability comparison in smoldering fire scene.

The determination of whether a fire has occurred is made possible by combining data on smoldering fires based on the traditional D-S evidence theory, the methods proposed by Murphy [23], Deng Y. [24], Deng. Z. [30], and Wang [33] and this paper, respectively. The method proposed in this paper can detect the occurrence of fire at t = 10 s, which is earlier than the 11.5 s of Wang’s method, 11.5 s of Deng Y.’s method, 12 s of Deng Z’s method, 13.5 s of traditional evidence theory, and 17 s of Murthy’s method, as shown in Figure 11. As illustrated in Figure 11, when compared to the traditional evidence theory, classical improvement method, and similar improvement method, the method proposed in this paper not only detects the occurrence of fire in advance, but also has a higher detection accuracy.

To further verify the effectiveness of the proposed fire detection method, we obtained different CO concentration, smoke concentration and temperature data by setting different combustibles, combustion locations, heat release rates, and heat ramp-up times. Then we made our own fire dataset, which included 1000 positive samples and 1000 negative samples. Based on the traditional evidence theory, classical improvement method, similarity improvement method and the proposed method in this paper, the homemade samples are fused to calculate the accuracy rate and false alarm rate of detection. Assuming that TP represents the number of samples correctly judged to be fires, FN represents the number of samples not correctly judged to be fires, FP represents the number of samples misreported to be fires, and TN represents the number of samples correctly judged to be fires that did not occur. The accuracy and false alarm rates (FAR) are calculated as Equation (22):

(22) $$\{\begin{array}{c} accurary|=\frac{TP+TN}{TP+FN+FP+TN}\\ FAR|=\frac{FP}{FP+TN} \end{array}.$$

Table 11 shows the fire detection accuracy and false alarm rate of various methods. According to Table 11, compared to other methods, the proposed method increased the fire detection rate by 0.7–10.2% and reduces the false alarm rate by 0.9–6.4%, which improves the reliability of fire discrimination obviously.

It is evident that when applied to indoor fire detection, the proposed heterogeneous data-fusion method has better fire detection performance and can simultaneously improve the timeliness and accuracy of detection, proving its feasibility and effectiveness in multi-sensor data fusion.

## 5. Conclusions

In this paper, a multi-sensor heterogeneous data fusion strategy based on the cloud model and improved evidence theory is presented, which can better cope with the ambiguity and conflict of heterogeneous multi-sensor gathered data. The cloud model is used to estimate the BPA function of each data source’s associated evidence. Evidence similarity is calculated by using multi-relationship measures, evidence certainty is measured by using interval distance, the body of evidence is jointly improved by combining the evidence’s similarity and certainty, and the improved body is fused by using Dempster’s rule. The usefulness of the improved evidence theory technique is validated in this research, and the results show that the proposed method performs better when dealing with both conflicting and normal evidence. The method is used for indoor fire detection in light of the issues of prolonged duration and low accuracy. Compared to traditional evidence theory, classical improvement method, and similar improvement method, the proposed method improves detection speed by 0.5–3 s, accuracy by 0.7–10.2%, and reduces the false alarm rate by 0.9–6.4%, which has better detection performance. It also provides a specific reference value for multi-source information fusion.

In future work, we intend to test the feasibility of the proposed method on other multi-sensor acquisition information systems, as well as investigate how to combine homogeneous and heterogeneous data fusion algorithms to fully exploit effective data information and improve data fusion accuracy.

## Figures and Tables

**Table 1:** Distribution of different bodies of evidence in different situations.

Situation	The Distribution of Evidence Body
Situation 1	$m_{1}\left(a\right)=m_{1}\left(b\right)=m_{1}\left(c\right)=m_{1}\left(d\right)=0.25$ $m_{2}\left(a\right)=m_{2}\left(b\right)=m_{2}\left(c\right)=m_{2}\left(d\right)=0.25$
Situation 2	$m_{1}\left(a\right)=m_{1}\left(b\right)=0.5$ $m_{2}\left(c\right)=m_{2}\left(d\right)=0.5$
Situation 3	$m_{1}\left(a\right)=m_{1}\left(b\right)=m_{1}\left(c\right)=1/3$ $m_{2}\left(a,b,c\right)=1$
Situation 4	$m_{1}\left(a\right)=0.25,m_{1}\left(b\right)=0.65,m_{1}\left(abc\right)=0.1$ $m_{2}\left(a\right)=0.65,m_{2}\left(b\right)=0.25,m_{2}\left(abc\right)=0.1$

**Table 2:** Four common conflicting BPA functions.

Types of Conflict	Evidences	Proposition BPA				
		*A*	*B*	*C*	*D*	*E*
Complete conflict (k = 1)	*m*_1_	1	0	0	\	\
	*m*_2_	0	1	0	\	\
	*m*_3_	0.8	0.1	0.1	\	\
	*m*_4_	0.8	0.1	0.1	\	\
0 trust conflict (k = 0.99)	*m*_1_	0.5	0.2	0.3	\	\
	*m*_2_	0.5	0.2	0.3	\	\
	*m*_3_	0	0.9	0.1	\	\
	*m*_4_	0.5	0.2	0.3	\	\
1 trust conflict (k = 0.9998)	*m*_1_	0.9	0.1	0	\	\
	*m*_2_	0	0.1	0.9	\	\
	*m*_3_	0.1	0.15	0.75	\	\
	*m*_4_	0.1	0.15	0.75	\	\
High conflict (k = 0.9999)	*m*_1_	0.7	0.1	0.1	0	0.1
	*m*_2_	0	0.5	0.2	0.1	0.2
	*m*_3_	0.6	0.1	0.15	0	0.15
	*m*_4_	0.55	0.1	0.1	0.15	0.1
	*m*_5_	0.6	0.1	0.2	0	0.1

**Table 3:** Similarity and certainty of each evidence under four conflicts.

Types of Conflict	Evidences	Global Similarity $s_{i}$	Determinacy $DU\left(m_{i}\right)$
Complete conflict	*m*_1_	1.628	3
	*m*_2_	0.036	3
	*m*_3_	1.832	4.527
	*m*_4_	1.832	4.527
0 trust conflict	*m*_1_	2.141	6.009
	*m*_2_	2.141	6.009
	*m*_3_	0.423	3.785
	*m*_4_	2.141	6.009
1 trust conflict	*m*_1_	0.068	3.785
	*m*_2_	1.715	3.785
	*m*_3_	1.891	4.858
	*m*_4_	1.891	4.858
High conflict	*m*_1_	2.651	7.194
	*m*_2_	0.631	8.127
	*m*_3_	2.737	7.733
	*m*_4_	2.779	7.987
	*m*_5_	2.888	7.718

**Table 4:** Fusion results of four common conflicts.

Types of Conflict	Methods	Proposition					Θ
		*A*	*B*	*C*	*D*	*E*	
	D-S	\	\	\	\	\	Invalid
Complete conflict	Sun	0.0917	0.0423	0.0071	\	\	0.8589
	Murthy	0.8204	0.1748	0.0048	\	\	0
	Deng Y.	0.8166	0.1164	0.0670	\	\	0
	Deng Z.	0.9792	0.0207	0.0001	\	\	0
	Wang	0.9996	0.0002	0.0002	\	\	0
	This paper	0.9999	0.0001	0.0001	\	\	0
	D-S	0	0.7270	0.2730	0	0	0
0 trust conflict	Sun	0.0525	0.0597	0.0377	\	\	0.8501
	Murthy	0.4091	0.4091	0.1818	\	\	0
	Deng Y.	0.4318	0.2955	0.2727	\	\	0
	Deng Z.	0.6510	0.2384	0.1106	\	\	0
	Wang	0.7628	0.2200	0.0172	\	\	0
	This paper	0.8421	0.0428	0.1151	\	\	0
	D-S	0	1	0	0	0	0
1 trust conflict	Sun	0.0388	0.0179	0.0846	\	\	0.8587
	Murthy	0.1676	0.0346	0.7978	\	\	0
	Deng Y.	0.1388	0.1318	0.7294	\	\	0
	Deng Z.	0.0273	0.0018	0.9709	\	\	0
	Wang	0.0006	0.0015	0.9980	\	\	0
	This paper	0.0001	0.0008	0.9991	\	\	0
	D-S	0	0.3571	0.4286	0	0.2143	0
High conflict	Sun	0.0443	0.0163	0.0163	0.0045	0.0118	0.9094
	Murthy	0.7637	0.1031	0.0716	0.0080	0.0538	0
	Deng Y.	0.5324	0.1521	0.1462	0.0451	0.1241	0
	Deng Z.	0.9846	0.004	0.0055	0.0001	0.0029	0
	Wang	0.9911	0.0025	0.001	0.0	0.0004	0
	This paper	0.9983	0.0002	0.0013	0.0	0.0002	0

**Table 5:** Single focal element evidence body data distribution.

Evidences	*A*	*B*	*C*
*m*_1_	0.5	0.2	0.3
*m*_2_	0	0.8	0.2
*m*_3_	0.6	0.3	0.1
*m*_4_	0.55	0.25	0.2
*m*_5_	0.65	0.15	0.2

**Table 6:** Multi-focus evidence body data distribution.

Evidences	*A*	*B*	*C*	*AC*
*m*_1_	0.5	0.2	0.3	0
*m*_2_	0	0.9	0.1	0
*m*_3_	0.55	0.1	0	0.35
*m*_4_	0.55	0.1	0	0.35
*m*_5_	0.6	0.1	0	0.3

**Table 7:** Similarity and certainty of evidence under single and multifocal elements.

Evidences	$\mathrm{Global}\;\mathrm{Similarity}\;s_{i}$		$\mathrm{Determinacy}\;DU\left(m_{i}\right)$	
	Single-Focal Element	Multi-Focal Element	Single-Focal Element	Multi-Focal Element
*m*_1_	2.743	6.009	2.496	6.009
*m*_2_	0.858	4.485	0.345	3.785
*m*_3_	2.756	5.599	3.685	3.221
*m*_4_	2.983	5.868	3.685	3.221
*m*_5_	2.999	5.434	3.730	3.422

**Table 8:** Multi-quantity evidence body fusion results.

Methods	*m*_1_–*m_3_*		*m*_1_–*m*_4_		*m*_1_–*m*_5_	
	Single-Focal Element	Multi-Focal Element	Single-Focal Element	Multi-Focal Element	Single-Focal Element	Multi-Focal Element
D-S	*m(A)* = 0 *m(B)* = 0.9132 *m(C)* = 0.0868	*m(A)* = 0 *m(B)* = 0.6315 *m(C)* = 0.3685 *m(AC) =* 0	*m(A)* = 0 *m(B)* = 0.9293 *m(C)* = 0.0707	*m(A)* = 0 *m(B)* = 0.3287 *m(C)* = 0.6713 *m(AC) =* 0	*m(A)* = 0 *m(B)* = 0.9079 *m(C)* = 0.0921	*m(A)* = 0 *m(B)* = 0.1403 *m(C)* = 0.8597 *m(AC) =* 0
Murthy	*m(A)* = 0.3555 *m(B)* = 0.5868 *m(C)* = 0.0577	*m(A)* = 0.5568 *m(B)* = 0.3562 *m(C)* = 0.0782 *m(AC) =* 0.0088	*m(A)* = 0.5453 *m(B)* = 0.4246 *m(C)* = 0.0301	*m(A)* = 0.8656 *m(B)* = 0.0891 *m(C)* = 0.0382 *m(AC) =* 0.0074	*m(A)* = 0.8090 *m(B)* = 0.1785 *m(C)* = 0.0125	*m(A)* = 0.9688 *m(B)* = 0.0156 *m(C)* = 0.0127 *m(AC) =* 0.0029
Deng Y.	*m(A)* = 0.4978 *m(B)* = 0.4434 *m(C)* = 0.0588	*m(A)* = 0.6500 *m(B)* = 0.2547 *m(C)* = 0.0858 *m(AC)* = 0.0095	*m(A)* = 0.7418 *m(B)* = 0.2312 *m(C)* = 0.0270	*m(A)* = 0.9305 *m(B)* = 0.0274 *m(C)* = 0.0339 *m(AC)* = 0.0082	*m(A)* = 0.9277 *m(B)* = 0.0633 *m(C)* = 0.0090	*m(A)* = 0.9846 *m(B)* = 0.0024 *m(C)* = 0.0098 *m(AC)* = 0.0032
Deng Z.	*m(A)* = 0.6367 *m(B)* = 0.2631 *m(C)* = 0.1002	*m(A)* = 0.5669 *m(B)* = 0.3325 *m(C)* = 0.0966 *m(AC)* = 0.0044	*m(A)* = 0.6603 *m(B)* = 0.3095 *m(C)* = 0.0301	*m(A)* = 0.8389 *m(B)* = 0.1068 *m(C)* = 0.0507 *m(AC)* = 0.0036	*m(A)* = 0.8733 *m(B)* = 0.1152 *m(C)* = 0.0115	*m(A)* = 0.9136 *m(B)* = 0.0454 *m(C)* = 0.0357 *m(AC)* = 0.0053
Wang	*m(A)* = 0.6594 *m(B)* = 0.3119 *m(C)* = 0.0286	*m(A)* = 0.6581 *m(B)* = 0.2409 *m(C)* = 0.0937 *m(AC)* = 0.0073	*m(A)* = 0.8142 *m(B)* = 0.1604 *m(C)* = 0.0255	*m(A)* = 0.9391 *m(B)* = 0.0190 *m(C)* = 0.0342 *m(AC)* = 0.0077	*m(A)* = 0.9518 *m(B)* = 0.0401 *m(C)* = 0.0081	*m(A)* = 0.9859 *m(B)* = 0.0014 *m(C)* = 0.0096 *m(AC)* = 0.0031
This paper	*m(A)* = 0.7983 *m(B)* = 0.175 *m(C)* = 0.0267	*m(A)* = 0.8368 *m(B)* = 0.0478 *m(C)* = 0.1105 *m(AC)* = 0.0049	*m(A)* = 0.8842 *m(B)* = 0.0944 *m(C)* = 0.0221	*m(A)* = 0.9597 *m(B)* = 0.0028 *m(C)* = 0.0316 *m(AC)* = 0.0059	*m(A)* = 0.9849 *m(B)* = 0.0109 *m(C)* = 0.0026	*m(A)* = 0.9895 *m(B)* = 0.0003 *m(C)* = 0.0078 *m(AC)* = 0.0024

**Table 9:** Normal evidence body data distribution.

Evidences	*A*	*B*	*C*
*m*_1_	0.85	0.05	0.1
*m*_2_	0.70	0.15	0.15
*m*_3_	0.50	0.20	0.30
*m*_4_	0.50	0.20	0.30
*m*_5_	0.7	0.25	0.05

**Table 10:** Normal evidence fusion result.

Methods	*m(A)*	*m(B)*	*m(C)*
D-S	0.9985	0.0007	0.0008
This paper	1	0	0

**Table 11:** Comparison of fire detection accuracy and false alarm rate of different methods.

Fusion Methods	Accuracy Rate	False Alarm Rates
Traditional D-S	88.6%	7.2%
Murthy	93.4%	5.6%
Deng Y.	96.6%	2.2%
Deng Z.	96.3%	3.1%
Wang	98.1%	1.7%
The method proposed	98.8%	0.8%

## Data Availability

Not applicable.
